# Psychiatric Comorbidities Associated with Food Addiction in Post-Bariatric Patients: Toward Personalized Mental Health Screening and Postoperative Care

**DOI:** 10.3390/jpm15070313

**Published:** 2025-07-14

**Authors:** Ligia Florio, Maria Olivia Pozzolo Pedro, Kae Leopoldo, Maria Amalia Accari Pedrosa, João Mauricio Castaldelli-Maia

**Affiliations:** 1 Department of Psychiatry, Medical School, University of São Paulo, São Paulo 05403-903, Brazil; floriol@usp.br (L.F.);; 2 Department of Experimental Psychology, Psychology Institute, University of São Paulo, São Paulo 05508-030, Brazil; kae.leopoldo@usp.br; 3 Instituto Perdizes (IPER), Hospital das Clinicas HCFMUSP, Faculdade de Medicina, Universidade de Sao Paulo, São Paulo 05021-001, Brazil; m.amalia@hc.fm.usp.br; 4 Department of Epidemiology, Mailman School of Public Health, Columbia University, New York, NY 10032, USA

**Keywords:** food addiction, bariatric surgery, psychiatric disorders, comorbidity, mYFAS 2.0, mental health

## Abstract

**Background:** Food addiction (FA) is an emerging construct that mirrors the behavioral and neurobiological characteristics of substance use disorders. Despite growing interest, its association with specific psychiatric disorders among bariatric patients remains understudied. **Objective:** Our aim was to examine the prevalence and strength of associations between FA and seven major psychiatric disorders in individuals who underwent bariatric surgery. **Methods:** In a sample of 100 post-bariatric patients referred for psychiatric evaluation, FA was assessed using the modified Yale Food Addiction Scale 2.0 (mYFAS 2.0), and psychiatric disorders were diagnosed using the Mini International Neuropsychiatric Interview (MINI). Logistic regression models were used to estimate adjusted odds ratios (aORs) for the association between FA and each psychiatric disorder, controlling for sex, age, body mass index (BMI), employment status, the number of children, clinical comorbidities, physical activity, family psychiatric history, and region of residence. **Results:** FA was present in 51% of the sample. Descriptive analyses revealed a significantly higher prevalence of major depressive disorder, panic disorder, generalized anxiety disorder, social anxiety disorder, agoraphobia, obsessive–compulsive disorder, and bulimia nervosa among individuals with FA. Multivariate models showed robust associations between FA and bulimia nervosa (aOR = 19.42, *p* < 0.05), generalized anxiety disorder (aOR = 2.88, *p* < 0.05), obsessive–compulsive disorder (aOR = 6.64, *p* < 0.05), agoraphobia (aOR = 3.14, *p* < 0.05), social anxiety disorder (aOR = 4.28, *p* < 0.05) and major depressive disorder (aOR = 2.79, p < 0.05). **Conclusions:** FA is strongly associated with a range of psychiatric comorbidities in post-bariatric patients, reinforcing the need for comprehensive mental health screening in this population. These findings underscore the potential role of FA as a clinical marker for stratified risk assessment, supporting more personalized approaches to mental health monitoring and intervention following bariatric surgery.

## 1. Introduction

Obesity is a complex, multifactorial condition associated with substantial physical and mental health burdens [1,2]. Bariatric surgery is an effective treatment for morbid obesity, leading to significant weight loss and improvements in metabolic, cognitive, and psychological outcomes [3,4,5,6]. Despite these benefits, many patients continue to experience psychiatric disorders postoperatively, including depression, anxiety, and eating disorders [7,8].

Food addiction (FA) has emerged as a construct of growing clinical interest, characterized by compulsive overeating, cravings, and continued consumption despite adverse consequences—mirroring patterns observed in substance use disorders [9,10,11,12]. Studies suggest that FA affects 13–20% of post-bariatric patients [13,14] and is associated with emotional dysregulation, impulsivity, and compulsivity—these factors are linked to poorer surgical outcomes, including weight regain [15,16].

Psychiatric comorbidities are highly prevalent in the bariatric population, with estimates suggesting that up to 55% of patients present with psychiatric diagnoses preoperatively, which may persist or emerge after surgery [17,18,19]. FA frequently overlaps with bulimia nervosa, binge eating disorder, and anxiety disorders, reflecting shared features such as impaired impulse control and dysfunctional reward processing [15,20].

Despite this, the psychiatric profiles of post-bariatric patients with FA remain underexplored. Addressing this gap is crucial for developing personalized interventions that improve both psychological wellbeing and surgical outcomes [21,22]. This study aims to examine the prevalence and strength of associations between FA and a range of psychiatric disorders in individuals who have undergone bariatric surgery, contributing to more comprehensive and personalized mental health care within this population.

## 2. Materials and Methods

This cross-sectional study was conducted at the Psychiatry Outpatient Clinic of the Hospital Estadual Mário Covas (HEMC) in Santo André, São Paulo, Brazil. Participants were recruited from a specialized follow-up program for patients who had undergone bariatric surgery previously. Recruitment took place during psychiatric evaluations conducted between October 2019 and June 2022.

### 2.1. Sample and Procedure

Participants were included if they had undergone bariatric surgery at least 12 months before the assessment to allow for postoperative stabilization. No a priori sample size calculation was conducted; instead, a convenience sample of 100 patients was recruited based on eligibility and availability during the study period. All participants provided written informed consent before enrollment. Inclusion criteria were age ≥18 years, a prior Roux-en-Y gastric bypass or sleeve gastrectomy surgery, and the ability to complete structured interviews. Exclusion criteria included severe cognitive impairment, ongoing psychotic episodes, or refusal to participate. Severe cognitive impairment was defined as the inability to comprehend or complete structured interviews due to marked disorientation or communication difficulties. This was assessed through preliminary orientation and comprehension questions administered by trained interviewers before initiating the MINI.

Participants were interviewed in a private setting by trained researchers using validated instruments to collect sociodemographic, clinical, and psychiatric data. The protocol was approved by the Ethics Committees of both HEMC and Escola Paulista de Ciências Médicas (CAAE 17812619.7.0000.9027).

### 2.2. Measures

Food Addiction: FA was assessed using the Brazilian Portuguese version of the modified Yale Food Addiction Scale 2.0 (mYFAS 2.0), validated by Nunes-Neto et al. (2018) [23]. The mYFAS 2.0 is a 13-item self-report measure aligned with DSM-5 criteria for substance use disorders. A diagnostic threshold was established when participants met at least two of the symptom criteria and reported significant clinical impairment or distress.

Psychiatric Disorders: The diagnoses of current and lifetime psychiatric disorders were established using the Mini International Neuropsychiatric Interview (MINI), version 5.0.0, validated for the Brazilian population. The MINI provides structured assessments based on DSM-IV and ICD-10 criteria, covering major depressive disorder, anxiety disorders, obsessive–compulsive disorder, eating disorders, substance use disorders, and psychotic syndromes. While the MINI version 5.0.0 follows DSM-IV and ICD-10 criteria, it remains widely used in clinical and research settings for its brevity and reliability. However, certain diagnostic definitions—particularly regarding eating disorders—differ in DSM-5, which may impact the categorization and prevalence estimates of some conditions.

Clinical and Sociodemographic Variables: Data on age, sex, marital status, employment status, the number of children, place of residence, body mass index (BMI), post-surgical time (in months), physical activity level, and comorbid clinical conditions were collected through structured interviews. Data on the type of bariatric surgery conducted, weight loss, and weight regain were obtained from clinical records and participant reports.

### 2.3. Statistical Analysis

Descriptive analyses were used to characterize the total sample and compare participants with and without FA. Categorical variables were compared using Chi-square tests, and continuous variables were assessed using independent *t*-tests or Mann–Whitney U, depending on the distribution. Continuous variables were tested for normality using the Shapiro–Wilk test. As age, the number of children, BMI, current weight, and time since surgery did not follow a normal distribution (*p* < 0.05), we used the Wilcoxon rank-sum test (Mann–Whitney U) for comparisons between groups. Categorical variables were analyzed using the chi-square test or Fisher’s exact test, as appropriate. All tests were two-tailed, with a significance level set at 5%.

To examine associations between FA and psychiatric disorders, multivariate logistic regression models were constructed. Each model included FA as the primary independent variable and one psychiatric disorder as the dependent variable. Covariates included age, sex, BMI, employment status, the number of children, clinical comorbidities, physical activity, and geographic origin. Adjusted odds ratios (aORs) with 95% confidence intervals (CIs) were reported. All analyses were conducted using Stata^®^ version 16.1 (StataCorp, College Station, TX, USA). Statistical significance was set at *p* < 0.05.

## 3. Results

Among the 100 participants included in this study, 51 (51%) met the criteria for FA based on the mYFAS 2.0. Table 1 summarizes the sociodemographic characteristics of the sample. No significant differences were observed between the groups in terms of age, sex, or the number of children. However, individuals with FA were significantly more likely to reside outside the hospital’s regional area (*p* = 0.046) and to be unemployed (68.6% vs. 12.2%; *p* = 0.021).

Table 2 presents the clinical characteristics. There were no statistically significant differences in BMI, the percentage of excess weight loss, current weight, or post-surgical time. Likewise, clinical comorbidities, smoking status, alcohol use, family psychiatric history, and physical activity levels did not differ significantly between groups. These findings suggest that the presence of FA is not associated with distinct somatic or surgical profiles.

Psychiatric morbidity was considerably higher among participants with FA (Table 3). Statistically significant differences were found in the prevalence of major depressive disorder (current episode: 66.7% vs. 44.9%, *p* = 0.028), lifetime panic disorder (56.9% vs. 30.6%, *p* = 0.008), agoraphobia (68.6% vs. 40.8%, *p* = 0.005), generalized anxiety disorder (70.6% vs. 46.9%, *p* = 0.016), social anxiety disorder (25.5% vs. 8.2%, *p* = 0.021), obsessive–compulsive disorder (25.5% vs. 4.1%, *p* = 0.003), and bulimia nervosa (21.6% vs. 2.0%, *p* = 0.003). Other psychiatric conditions, such as PTSD, bipolar spectrum disorders, and psychotic symptoms, did not differ significantly between groups.

Multivariate logistic regression models (Table 4) confirmed that FA was independently associated with several psychiatric disorders. Adjusting for potential confounders, FA was strongly associated with bulimia nervosa (aOR = 19.42; 95% CI: 2.06–183.40; *p* = 0.010), OCD (aOR = 6.64; 95% CI: 1.32–33.49; *p* = 0.022), social anxiety disorder (aOR = 4.28; 95% CI: 1.16–15.79; *p* = 0.029), agoraphobia (aOR = 3.14; 95% CI: 1.23–8.05; *p* = 0.020), generalized anxiety disorder (aOR = 2.88; 95% CI: 1.17–7.09; *p* = 0.020), and major depressive disorder (aOR = 2.79; 95% CI: 1.09–7.12; *p* = 0.030). The association with lifetime panic disorder approached significance (aOR = 2.40; *p* = 0.060).

## 4. Discussion

This study contributes to a growing body of evidence suggesting that FA is not only prevalent among individuals who have undergone bariatric surgery but is also associated with a considerable psychiatric burden. Among our sample, over half of the participants met the diagnostic threshold for FA using the mYFAS 2.0, and this subgroup exhibited significantly higher rates of several psychiatric conditions, including major depressive disorder, generalized anxiety disorder, social anxiety disorder, agoraphobia, obsessive–compulsive disorder, and bulimia nervosa. These findings remained significant even after adjusting for sociodemographic and clinical covariates, including age, sex, BMI, employment status, the number of children, physical activity, region of residence, clinical comorbidities, and family psychiatric history, and support a precision medicine approach, suggesting that individuals with FA represent a distinct psychiatric phenotype among post-bariatric patients. Integrating FA assessment into routine care could help personalize postoperative psychiatric monitoring and interventions.

The observed FA prevalence of 51% in our sample is higher than that reported in previous studies, which ranges from 13% to 20% among post-bariatric patients [13,14]. The clinical setting of our sample may partially explain this discrepancy, as all participants were referred to a psychiatric outpatient clinic, potentially representing a population with more severe psychopathology. Alternatively, it could reflect broader diagnostic criteria captured by the mYFAS 2.0 or cultural and contextual factors specific to the Brazilian population.

Recent developments in the nosology of eating disorders have introduced constructs such as orthorexia nervosa—which is a condition characterized by an obsessive focus on healthy eating—and this also appears to share common psychological underpinnings with FA, particularly emotional dysregulation and compulsive behaviors [24]. These findings support the notion that emotional alterations are a transdiagnostic feature across traditional and emerging eating disorders, reinforcing the need for multidimensional assessment tools that capture the complexity of eating-related psychopathology in post-bariatric populations.

The most robust associations emerged between FA and bulimia nervosa, OCD, and generalized anxiety disorder. The link between FA and bulimia nervosa is well-documented and intuitive, given shared features such as a loss of control over eating, emotional dysregulation, and compulsive food behaviors [12,25]. However, the magnitude of this association in our regression model (aOR = 19.42) underscores the severity of overlap. It suggests that FA may be a critical clinical marker for identifying patients with subthreshold or undiagnosed eating disorders following bariatric surgery.

The strong association between FA and obsessive–compulsive disorder (aOR = 6.64) is also notable. This finding aligns with prior research suggesting that FA behaviors share characteristics with compulsivity, including intrusive thoughts about food and repetitive behaviors to reduce distress [26]. Neurobiological studies have identified overlapping circuits in reward processing and executive dysfunction between OCD and FA, including altered dopaminergic signaling in the cortico-striatal [11,27].

Mood and anxiety disorders were also significantly associated with FA in our study. Major depressive disorder, agoraphobia, social anxiety, and generalized anxiety disorder were all more prevalent among participants with FA. These findings reinforce prior evidence suggesting that affective dysregulation may be both a consequence and a driver of compulsive eating behaviors [16,28]. The link between depression and FA could be explained through the use of hyperpalatable food as a maladaptive coping mechanism, with transient mood relief reinforcing consumption despite long-term negative [15].

Importantly, these associations were not mediated by somatic or surgical characteristics. BMI, the percentage of weight loss, post-surgical time, and the presence of medical comorbidities did not differ between groups. This suggests that FA and its associated psychiatric morbidity represent a psychological and behavioral domain distinct from the physiological outcomes of bariatric surgery. Therefore, weight-related metrics alone may not fully capture patients’ recovery trajectories or quality of life. Although time since surgery was recorded and did not differ significantly between groups, it may nonetheless influence the development or persistence of FA and related psychiatric conditions. Previous research suggests that psychological adaptations after bariatric surgery evolve over time, with some disorders emerging or intensifying in later postoperative phases. Future studies should explore whether the time since surgery moderates the relationship between FA and psychiatric outcomes, potentially identifying critical windows for preventive interventions.

The clinical implications of these findings are multifold. First, they support the integration of routine psychiatric screening into bariatric follow-up protocols, particularly using validated tools such as the mYFAS 2.0. Second, the identification of FA could serve as a proxy for broader psychiatric vulnerability, justifying multidisciplinary interventions that address emotional regulation, trauma history, and eating behaviors simultaneously. Third, our findings highlight the limitations of a purely biomedical model for obesity management, emphasizing the need for biopsychosocial frameworks in both preoperative evaluations and long-term care. From a precision psychiatry perspective, FA may serve as a valuable clinical and behavioral biomarker, enabling the early identification of high-risk individuals and the personalization of supportive care to improve surgical and mental health outcomes.

From a public health perspective, the high prevalence of FA and psychiatric comorbidities in our sample underscores the need for greater investment in mental health services within bariatric programs. Given the chronic and relapsing nature of both addiction and mood disorders, ongoing psychosocial support may be essential to sustain the benefits of bariatric surgery and prevent weight regain or psychological decompensation.

Several limitations should be noted. The cross-sectional design of this study precludes causal inference, and the convenience sample drawn from a psychiatric outpatient clinic may limit the generalizability of the findings. Moreover, MINI is based on DSM-IV criteria and may not capture newer diagnostic entities or the full-dimensional spectrums of psychopathology. The use of the MINI based on DSM-IV criteria may also have influenced diagnostic outcomes, especially in domains like eating disorders, where DSM-5 introduced revised criteria. Consequently, some patients might not meet current diagnostic thresholds despite presenting clinically significant symptoms. Nonetheless, the use of validated instruments, structured interviews, and statistical adjustments strengthens the reliability of our results. Additionally, our recruitment strategy—limited to individuals referred to for psychiatric evaluation in a specialized outpatient clinic—likely contributed to an overrepresentation of psychiatric comorbidities. This may limit the generalizability of our findings to broader post-bariatric populations. Patients seeking or referred for mental health support may differ systematically from those in primary or surgical follow-up care, particularly in terms of emotional regulation, vulnerability to addictive behaviors, and help-seeking attitudes.

Future research should explore the longitudinal trajectory of FA and psychiatric comorbidities following surgery, incorporating neurobiological, behavioral, and social determinants. Studies with more diverse samples, including those recruited from community or primary care settings, should further clarify prevalence estimates and risk factors. Additionally, the randomized controlled trials of tailored psychological or pharmacological interventions for individuals with FA could evaluate efficacy and inform clinical guidelines. While our primary models did not explore interaction effects, future analyses could assess whether demographic variables—such as sex, age, or socioeconomic status—moderate the associations between FA and psychiatric outcomes. Identifying these moderating factors could support more refined patient stratification in personalized mental health care.

In conclusion, FA is highly prevalent in this psychiatric bariatric sample and is independently associated with a broad range of psychiatric disorders. These findings reinforce the need for integrated mental health care in bariatric pathways and suggest that FA may serve as a useful clinical marker for identifying patients at risk of poor psychological outcomes. Addressing this psychiatric burden is essential not only for enhancing individual recovery but also for optimizing the overall effectiveness and sustainability of obesity treatment strategies. Importantly, our findings contribute to the growing body of knowledge supporting precision medicine, where stratification based on behavioral and psychiatric phenotypes—such as FA—may enhance individualized care pathways for post-bariatric patients.

## Figures and Tables

**Table 1:** Sociodemographic characteristics of post-bariatric patients with and without food addiction (*n* = 100).

Variable	With FA (*n* = 51)	Without FA (*n* = 49)	*p*-Value
Age (mean ± SD)	41.94 ± 9.52	44.08 ± 11.34	0.481
Number of Children (mean ± SD)	1.49 ± 1.36	1.71 ± 1.95	0.773
Sex			0.826
- Male	7 (13.7%)	6 (12.2%)	
- Female	44 (86.3%)	43 (87.8%)	
City of Residence			0.212
- Hospital’s city ^#^	11 (21.6%)	16 (32.7%)	
- Other cities	40 (78.4%)	33 (67.3%)	
Region of Residence			0.046 *
- Hospital region ^&^	20 (39.2%)	29 (59.2%)	
- Other regions	31 (60.8%)	20 (40.8%)	
Working Status			0.021 *
- Employed	16 (31.4%)	43 (87.8%)	
- Unemployed	35 (68.6%)	6 (12.2%)	
Marital Status			0.537
- Single	13 (25.5%)	12 (24.5%)	
- Married	35 (68.6%)	31 (63.3%)	
- Previously married	3 (5.9%)	6 (12.2%)	

^#^ Refers to Santo André, São Paulo, where the hospital is located. ^&^ The hospital’s region corresponds to the Greater ABC metropolitan area. * Statistically significant results (*p* < 0.05).

**Table 2:** Clinical characteristics of post-bariatric patients with and without food addiction (*n* = 100).

Variable	With FA (*n* = 51)	Without FA (*n* = 49)	*p*-Value
% Excess Weight Loss (mean ± SD)	49.0 ± 24.0	49.0 ± 36.0	0.644
Body Mass Index (BMI, mean ± SD)	35.22 ± 6.02	35.18 ± 8.55	0.427
Post-Surgical Time (months, mean ± SD)	56.27 ± 40.95	66.91 ± 41.48	0.098
Current Weight (kg, mean ± SD)	98.51 ± 24.76	96.99 ± 22.89	0.803
Any Clinical Diseases	29 (54.7%)	24 (45.3%)	0.430
Any Allergies	13 (43.3%)	17 (56.7%)	0.315
Family Psychiatric History	26 (48.2%)	28 (51.8%)	0.537
Smoking Status			0.636
- Current Smoker	3 (5.9%)	5 (10.2%)	
- Former Smoker	4 (7.8%)	5 (10.2%)	
- Never Smoker	44 (86.3%)	39 (79.6%)	
Alcohol Use			0.325
- None	31 (60.8%)	33 (67.3%)	
- Moderate Use	12 (23.5%)	6 (12.2%)	
- Abuse	8 (15.7%)	10 (20.4%)	
Physical Activity			0.896
- Active	15 (29.4%)	15 (30.6%)	
- Inactive	36 (70.6%)	34 (69.4%)	
Weight Regain			0.847
- 0–5%	6 (11.8%)	5 (10.2%)	
- 5–10%	16 (31.4%)	18 (36.7%)	
- >10%	29 (56.9%)	26 (53.1%)	
Surgical Technique			0.809
- Roux-en-Y Gastric Bypass	25 (49.0%)	24 (49.0%)	
- Sleeve Gastrectomy	3 (5.9%)	2 (4.1%)	
- Y-de-Roux Variant	12 (23.5%)	9 (18.4%)	
- Mixed/Others	11 (21.6%)	14 (28.6%)	

Notes: Data are presented as the mean ± standard deviation or *n* (%).

**Table 3:** Prevalence of psychiatric disorders among post-bariatric patients with and without food addiction (*n* = 100).

Psychiatric Disorder	With FA (*n* = 51)	Without FA (*n* = 49)	*p*-Value
Major Depressive Episodes (Current)	34 (66.7%)	22 (44.9%)	0.028 *
Major Depressive Episodes (Recurrent)	19 (37.3%)	18 (36.7%)	0.957
Depressive Episodes with Melancholic Features	25 (49.0%)	16 (32.6%)	0.096
Dysthymia (Current)	0 (0.0%)	3 (6.1%)	0.073
Suicide Risk (Any Level)	24 (47.1%)	17 (34.7%)	0.209
- Low Risk	16 (31.4%)	10 (20.4%)	0.211
- Medium Risk	1 (2.0%)	1 (2.0%)	0.977
- High Risk	7 (13.7%)	6 (12.2%)	0.826
Manic Episodes (Current)	12 (23.5%)	9 (18.4%)	0.526
Manic Episodes (Past)	14 (27.5%)	11 (22.4%)	0.564
Hypomanic Episodes (Current)	5 (9.8%)	4 (8.2%)	0.774
Hypomanic Episodes (Past)	5 (9.8%)	8 (16.3%)	0.332
Panic Disorder (Lifetime)	29 (56.9%)	15 (30.6%)	0.008 *
Panic Disorder (Current)	10 (19.6%)	9 (18.4%)	0.874
Agoraphobia (Current)	35 (68.6%)	20 (40.8%)	0.005 *
Social Anxiety Disorder (Current)	13 (25.5%)	4 (8.2%)	0.021 *
Obsessive–Compulsive Disorder (Current)	13 (25.5%)	2 (4.1%)	0.003 *
Post-Traumatic Stress Disorder (Current)	6 (11.8%)	5 (10.2%)	0.803
Alcohol Dependence (12 months)	9 (17.6%)	5 (10.2%)	0.284
Alcohol Abuse (12 months)	1 (2.0%)	1 (2.0%)	0.970
Other Substance Dependence	1 (2.0%)	0 (0.0%)	0.325
Other Substance Abuse	1 (2.0%)	0 (0.0%)	0.325
Psychotic Syndrome (Current)	4 (7.8%)	1 (2.0%)	0.183
Psychotic Syndrome (Lifetime)	10 (19.6%)	8 (16.3%)	0.669
Mood Disorder with Psychotic Features (Current)	1 (2.0%)	0 (0.0%)	0.325
Mood Disorder with Psychotic Features (Lifetime)	8 (15.7%)	5 (10.2%)	0.415
Bulimia Nervosa (Current)	11 (21.6%)	1 (2.0%)	0.003 *
Generalized Anxiety Disorder (Current)	36 (70.6%)	23 (46.9%)	0.016 *
Antisocial Personality Disorder (Lifetime)	4 (7.8%)	1 (2.0%)	0.183

Notes: FA: food addiction; *p*-values were calculated using Chi-square or Fisher’s exact test. * Statistically significant values (*p* < 0.05). Data are presented as *n* (%).

**Table 4:** Multivariate logistic regression models for psychiatric disorders associated with food addiction.

Psychiatric Disorder	Adjusted OR (95% CI)	*p*-Value
Major Depressive Disorder	2.79 (1.09–7.12)	0.030 *
Panic Disorder (Lifetime)	2.40 (0.96–5.99)	0.060
Agoraphobia	3.14 (1.23–8.05)	0.020 *
Generalized Anxiety Disorder	2.88 (1.17–7.09)	0.020 *
Social Anxiety Disorder	4.28 (1.16–15.79)	0.029 *
Obsessive–Compulsive Disorder	6.64 (1.32–33.49)	0.022 *
Bulimia Nervosa	19.42 (2.06–183.40)	0.010 *

Model Covariates: All regression models were adjusted for the following variables: sex, age, body mass index (BMI), employment status, the number of children, region of residence (hospital region vs. other), clinical comorbidities, family psychiatric history, and physical activity status. Notes: aOR: adjusted odds ratio; CI: confidence interval. * Statistical significance at *p* < 0.05. The dependent variable in each model is the presence of a psychiatric disorder. Main predictor: food addiction status (YFAS 2.0 diagnostic threshold).

## Data Availability

All the data created and developed for this study are presented in the manuscript.
